# Perioperative management in pediatric day surgery: a synthesis of best evidence

**DOI:** 10.3389/fped.2026.1741673

**Published:** 2026-03-27

**Authors:** Chunmei Chen, Lili Liu, Wenlong Lu, Mingqi Peng

**Affiliations:** 1 Department of Anesthesiology, Children’s Hospital of Nanjing Medical University, Nanjing, Jiangsu, China; 2 Department of Emergency, Children’s Hospital of Nanjing Medical University, Nanjing, Jiangsu, China; 3 Nursing Department, Children’s Hospital of Nanjing Medical University, Nanjing, Jiangsu, China

**Keywords:** evidence synthesis, evidence-based practice, guideline, pediatric day surgery, perioperative management

## Abstract

**Background:**

To retrieve, analyze, and extract evidence related to perioperative management in pediatric day surgery, and provide evidence-based foundation for clinical perioperative care of children undergoing day surgery.

**Methods:**

By browsing the websites of the national and international guideline databases, professional association websites, and several databases, including BMJ Best Practice, PubMed, Web of Science, Embase, CINAHL, Cochrane Library, CNKI, VIP Database, Wanfang Database, and the China Biomedical Literature Database, relevant literatures, guidelines, evidence summaries, meta-analyses, expert consensuses, systematic reviews, and randomized controlled trials (RCTs) about perioperative management in pediatric day surgery. The search period spanned from the inception of the databases to August 2024.

**Results:**

After initially identifying 1821 articles, 15 articles were ultimately included following the exclusion of literature that did not meet the standards, comprising 3 clinical decision-making articles, 4 guidelines, 4 systematic reviews, 3 expert consensus statements, and 1 randomized controlled trial. Evidence summarization was conducted from three aspects: construction of day surgery systems, quality and safety, and perioperative management, summarizing 26 pieces of best evidence.

**Conclusion:**

This evidence synthesis translates 26 best-evidence recommendations into an implementable roadmap for pediatric day-surgery teams, supporting context-adapted perioperative management to promote knowledge translation into practice.

## Background

Day surgery is an efficient healthcare delivery model and is particularly suitable for many pediatric procedures because children often have fewer chronic comorbidities and undergo shorter, lower-grade operations with rapid recovery (1, 2). However, perioperative recommendations for pediatric day surgery remain fragmented across disciplines and sources (e.g., anesthesia, surgery, nursing, and management), The guideline published by de Luca U et al. only covered common conditions suitable for pediatric day surgery and health assessments of the children (2), while the study by Rohi A et al. focused primarily on post-discharge management (3). This fragmentation limits consistent uptake in routine practice and may contribute to avoidable variation in safety and efficiency.

This challenge is increasingly urgent as pediatric day surgery continues to expand. European and American countries report that 60%–80% of pediatric surgeries are conducted as day procedures (4). In China, the proportion of elective pediatric surgeries performed as day procedures was only 10%–20% prior to 2020 (5), but leading children's hospitals have reported rapid growth (e.g., approximately 40% in 2023 in a major children's hospital in Nanjing). Rapid scale-up—especially in developing settings—heightens the need for standardized, implementable perioperative pathways to support safety, quality monitoring, caregiver preparedness, and post-discharge continuity.

Therefore, guided by the PIPOST framework (6), this study aimed to synthesize best evidence for perioperative management in pediatric day surgery and translate the evidence into actionable recommendations for clinical teams. The target population was children undergoing day surgery (recommended age range: 3 months to 18 years; for former preterm infants, post-conceptual age >60 weeks). Interventions covered perioperative management strategies including system construction, quality and safety measures, and full-process perioperative management. Professionals included surgeons, anesthesiologists, administrators, and auxiliary staff; outcomes included same-day cancellations, unplanned delays, delayed discharges, unplanned readmissions/reoperations, complications/adverse reactions, and satisfaction of children and families across relevant hospital settings.

## Methods

This systematic review was conducted and reported in accordance with the Preferred Reporting Items for Systematic Reviews and Meta-Analyses (PRISMA) 2020 guidelines.

### Retrieval strategy

The core project team independently performed comprehensive literature searches based on the “6S” model of the evidence pyramid, using a combination of subject terms and free terms in a top-down approach. The searches were conducted to identify relevant evidence on perioperative management in pediatric day surgery, ensuring a systematic and thorough review of the available literature. The databases searched for English-language literature were UpToDate, Joanna Briggs Institute (JBI) Evidence-Based Healthcare Center Database, International Guideline Network, U.S. National Guideline Clearinghouse, Scottish Intercollegiate Guidelines Network, National Institute for Health and Care Excellence (NICE), BMJ Best Practice, Cochrane Library, PubMed, Web of Science, CINAHL, and websites of professional organizations such as the Registered Nurses’ Association of Ontario (RNAO), American Academy of Pediatrics, Canadian Pediatric Society, Royal Australasian College of Physicians, Royal College of Pediatrics and Child Health. The Chinese-language literature was searched in Medlive Guidelines, the Chinese Medical Association Pediatrics Branch website, CNKI, Wanfang Database, VIP Database, and the Chinese Biomedical Literature Database. The search period spanned from the inception of the databases to August 2024. The searches were designed to include a wide range of evidence types, such as clinical guidelines, systematic reviews, expert consensus documents, and randomized controlled trials.

For English searches, using PubMed as an example, the search expressions were: (((children[MeSH Terms] OR child*[Title/Abstract] OR pediatric∗[Title/Abstract] OR infant*[Title/Abstract] OR baby*[Title/Abstract] OR youth*[Title/Abstract] OR toddler*[Title/Abstract] OR Adolescent*[Title/Abstract] OR teen*[Title/Abstract] OR preschooler*[Title/Abstract] OR [hospital, pediatric]) AND (ambulatory surgical procedure*[MeSH Terms] OR Ambulatory Surgical Procedure*[Title/Abstract] OR Outpatient surgery[Title/Abstract] OR day surgery[MeSH Terms] OR day Surgery[Title/Abstract] OR ambulatory surgery[Title/Abstract] OR ambulatory surgery center[Title/Abstract] OR day-case surgery[Title/Abstract] OR outpatient surgery [Title/Abstract] OR same day surgery[Title/Abstract])) AND (care[Title/Abstract] OR nurs*[Title/Abstract] OR activities, educational[MeSH Terms] OR education[Title/Abstract] OR activities, educational[MeSH Terms] OR manag*[Title/Abstract] OR accident prevention[MeSH Terms] OR prevent[Title/Abstract])) AND (guideline[MeSH Terms] OR Guid*[Title/Abstract] OR consensus[Title/Abstract] OR “systematic review “[Title/Abstract] OR “meta-analysis"[Title/Abstract] OR “randomized controlled trial"[Title/Abstract] OR recommendation*[Title/Abstract] OR evidence[Title/Abstract]).

### Inclusion and exclusion criteria

The inclusion and exclusion criteria were established using the PIPOST model. The inclusion criteria were: (1) study subjects were children undergoing day surgery; (2) the content of studies was related to perioperative management; (3) study types that included clinical decisions, guidelines, expert consensuses, evidence summaries, systematic reviews, meta-analyses, and randomized controlled trials; and (4) studies published in either Chinese or English. The exclusion criteria were: (1) duplicate publications or translated versions; (2) outdated guidelines with newer versions available; (3) incomplete information or unavailable full text; and (4) guideline interpretations.

### Literature screening process

Two project team members independently conducted the literature screening process. Initially, the screening was based on titles and abstracts. Full texts of the selected articles were then reviewed to confirm final inclusion. In instances where disagreements arose between the two reviewers, a third researcher was consulted to resolve the dispute through discussion. An ‘outdated guideline’ was defined as one for which a newer version had been published by an authoritative body (e.g., a professional society or government health department) that explicitly stated it was an update, revision, or replacement of the previous version. For instance, our study included the Guidelines for Day-Case Surgery 2019 by Bailey et al. (7), which superseded the previous relevant guidelines from the Association of Anaesthetists. Consequently, any version published before 2019 that was replaced by this new guideline was considered ‘outdated’ and excluded from our analysis.

### Risk of bias in included studies

All appraisals were conducted by reviewers trained in evidence-based methodology. For evidence summaries, quality assessments were traced back to the original literature, and the choice of evaluation tools was selected based on the type of original literature. When evaluation disagreements occurred, the head of the hospital's evidence-based nursing group was consulted for arbitration.

Three articles from UpToDate were directly included in the present study. Four guidelines were independently evaluated by four project team members using the 2017 version of the Appraisal of Guidelines for Research and Evaluation Instrument II (AGREE II) (8), which consists of 23 items across 6 domains. Each item was rated on a scale of 1 to 7, with 1 being “Strongly Disagree” and 7 being “Strongly Agree”. The final score for each domain was calculated as a standardized percentage, based on the formula: (Obtained score - Minimum possible score)/(Maximum possible score - Minimum possible score) × 100%. Based on the final AGREE II domain scores, guidelines were classified into three grades: Grade A for domain scores > 60%, Grade B ≥ 3 domains with scores ≥30%, and Grade C: ≥3 domains with scores < 30%, which were not recommended for use. The intraclass correlation coefficient (ICC) was used to assess the reliability and reproducibility of evaluators, validating the consistency of evaluation results. An ICC of 0.40–0.59 was considered fair, 0.60−0.79 was good, and values ≥ 0.8 was excellent.

Three expert consensus statements were evaluated using the 2017 Joanna Briggs Institute (JBI) Expert Opinion tool and methodological criteria for expert consensus articles. Six items were assessed, and each item was rated as “Yes,” “No,” “Unclear,” or “Uncertain” to determine selection bias (6). An additional 4 systematic reviews were evaluated using the 2017 version of the JBI AMSTAR2 tool to assess their methodological quality across 16 items. Each item was rated as “Yes,” “Partial Yes,” or “No” depending on the degree of compliance with the evaluation criteria. Special emphasis was placed on whether there was methodological bias in critical items, and the overall quality of systematic reviews was graded as “High,” “Moderate,” “Low,” or “Very Low” (6).

One randomized controlled trial by Akkoyun S et al. (9) was evaluated using the JBI Critical Appraisal Checklist for Randomized Controlled Trials, which includes 13 items. In that study, all items were evaluated as “Yes” except for item 5, “Was blinding of those delivering treatment (care providers) achieved?” and item 6, “Was blinding of outcome assessors achieved?”, which were marked as “Unclear”. After further discussion, the overall quality of the literature was deemed to be high, and the study was included in this analysis.

### Evidence extraction, integration, and summarization

The core project team independently extracted relevant evidence from the included literature, ensuring accurate capture of source information. The core project team conducted literature searching, screening, methodological quality appraisal, evidence extraction, and evidence integration/grading.

To ensure clarity and contextual usability of the recommendations, an independent panel of 17 pediatric-related experts (medical, nursing, and health management) reviewed only the translated Chinese wording for language clarity, terminology accuracy, and cultural applicability. The expert panel did not participate in methodological appraisal, evidence extraction decisions, or evidence integration/grading.

The process of evidence summarization followed several guiding principles. First, evidence with similar or complementary themes was combined. For example, “Day surgery should be equipped with medical resources necessary for day surgery, including equipment and facilities” (10) and “Day surgery requires necessary anesthesia monitoring facilities” (11) were merged into “necessary equipment and facilities.” Second, in cases where contradictory evidence was encountered, priority was given to higher-quality and more recently published evidence. For instance, two recommendations regarding children suitable for day surgery were identified: “Children suitable for day surgery and anesthesia should generally conform to American Society of Anesthesiologists (ASA) Class I or II; ASA Class III or IV children are not recommended for day surgery" (11) and “Children suitable for day surgery and anesthesia should generally conform to ASA Class I or II, and some Class III without significant cardiopulmonary disease and no surgical contraindications in preoperative examinations" (7). Both recommendations were published in 2019 and were rated “B” in the degree of recommendation. However, the latter scored ≥ 60% in 5 domains using AGREE II comprehensive evaluation, while the former scored ≥ 60% in only 2 domains. Therefore, the higher-quality recommendation from the latter source was selected. Lastly, independent items retained their original expressions.

All evidence was processed using the Joanna Briggs Institute Levels of Evidence and Grades of Recommendation system (2014 version) (6). The effectiveness, feasibility, appropriateness, and clinical significance of the evidence were evaluated to form recommendations, classified as either strong recommendations (Grade A) or weak recommendations (Grade B). For conflicts in evidence integration and grading evaluation, the project's evidence-based group members engaged in discussions to reach a consensus. This study was a literature review that did not involve human research, so ethical review was not required.

## Results

### Literature screening process and general characteristics of included literature

**Figure 1:**
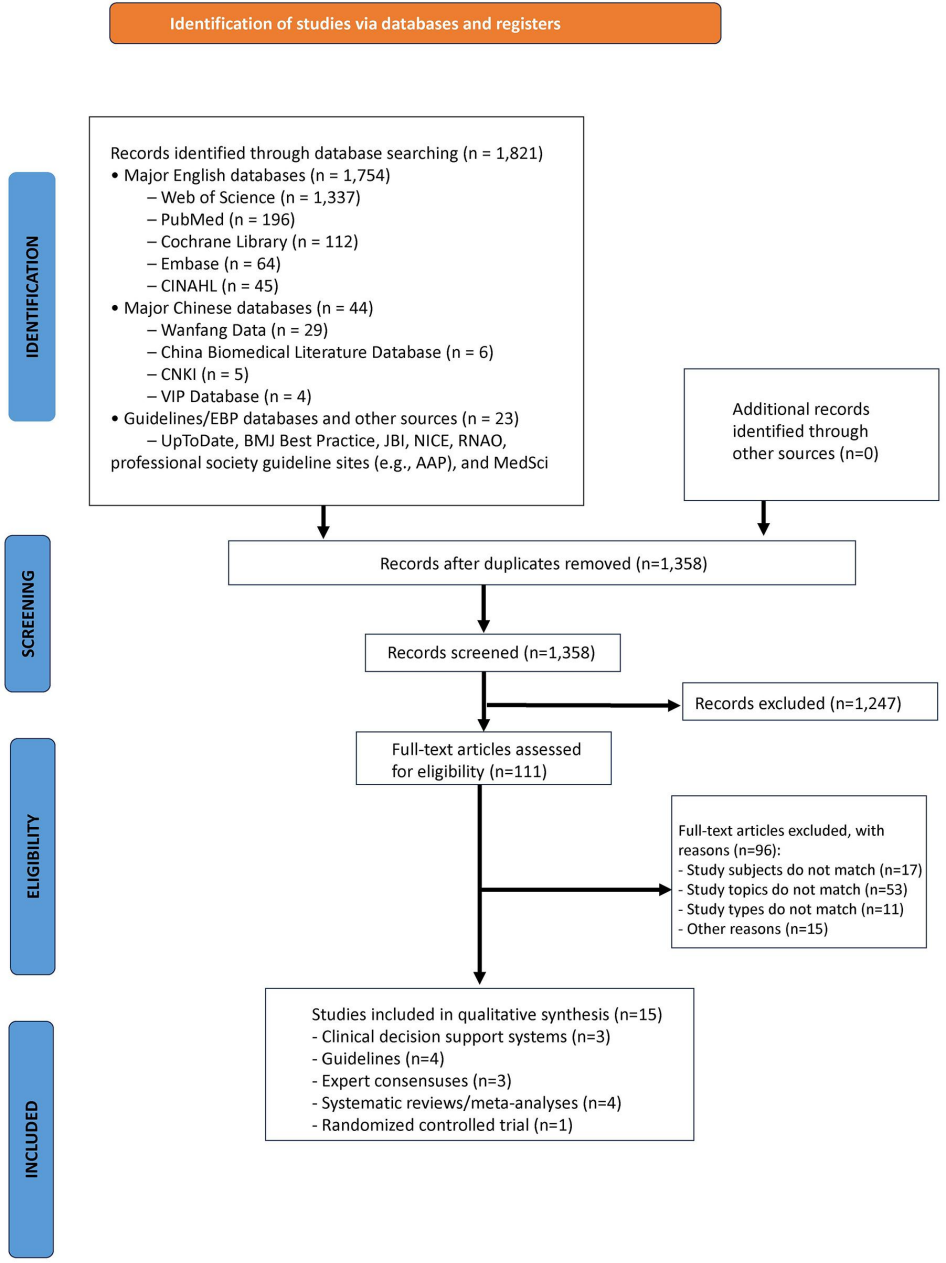
Literature screening process flow chart.

A total of 1,821 articles were retrieved through computer decision support systems and various databases. After removing duplicates, 1,358 articles remained. Following the review of titles and abstracts, 111 articles were preliminarily selected for full-text analysis. After a thorough reading of the full texts, 15 articles were ultimately included in this study (2, 7, 9–21). The included literature comprised 3 clinical decision support systems (12–14), 4 guidelines (2, 7, 10, 11), 3 expert consensuses (15–17), 4 meta-analyses and systematic reviews (18–21), and 1 randomized controlled trial (9). The literature screening process is shown in Figure 1, and the general characteristics of the included literature are presented in Table 1.

**Table 1:** General characteristics of included literature (*n* = 15).

Included Literature	Literature Source	Literature Type	Literature Topic	Publication/ Update Year
Schechter W. (12)	UpToDate	Clinical Decision	Management of acute perioperative pain in infants and children	2024
Shapiro FE et al. (13)	UpToDate	Clinical Decision	Anesthesia outpatient clinic	2024
SA Black et al. (14)	UpToDate	Clinical Decision	Drugs and techniques for general anesthesia in neonates and children	2024
Chinese Society of Cardiovascular Anesthesiology Day Surgery Anesthesia Branch (11)	MedSci	Guideline	Pediatric day surgery anesthesia guideline	2019
CR Bailey (7)	American Academy of Pediatrics	Guideline	2019 Day Surgery Guideline	2019
Chinese Society of Anesthesiology (10)	VIP Database	Guideline	Day surgery anesthesia guideline	2023
de Luca et al. (2)	Italian Society of Pediatric Surgery	Guideline	Italian Society of Pediatric Surgery and Pediatric Anesthesiology Society pediatric day surgery guideline	2018
Chinese Society of Anesthesiology (15)	CNKI	Expert Consensus	Day surgery anesthesia expert consensus	2016
Chinese Society of Pediatric Surgery Endoscopic Surgery Group et al. (16)	VIP Database	Expert Consensus	Pediatric surgery day surgery expert consensus	2020
National Clinical Research Center for Geriatric Diseases et al. (17)	VIP Database	Expert Consensus	Chinese expert consensus on operation and management of day surgery units in general hospitals	2022
Liu Shihui et al. (18)	Wanfang Database	Systematic Review	Meta-analysis of the effectiveness of enhanced recovery nursing in reducing postoperative complications in pediatric day surgery in China	2023
Liu Qian et al. (19)	CNKI	Systematic Review	Meta-analysis of temporary cancellation rates in day surgery	2024
Rantala A et al. (20)	PubMed	Systematic Review	Effectiveness of web-based mHealth interventions in pediatric outpatient surgery: A systematic review and meta-analysis of randomized controlled trials	2020
Kerimaa H et al. (21)	PubMed	Systematic Review	Effectiveness of preoperative interventions for day surgery in preschool children and their parents: A systematic review and meta-analysis of randomized controlled trials	2023
Akkoyun S et al. (9)	PubMed	Randomized Controlled Trial	Effect of perioperative written materials on anxiety levels of parents and home care of pediatric outpatient surgery patients: A randomized controlled trial	2023

### Quality assessment results of included literature

#### Quality assessment results of the included guidelines

For the 4 guidelines (2, 7, 10, 11), the intraclass correlation coefficient (ICC) was 0.817, indicating high consistency in the evaluation of these documents. Details are presented in Table 2.

**Table 2:** The quality evaluation results of the included guidelines (*n* = 4).

Included Literature	Standardized Percentage for Each Domain (%)						Overall Assessment(n)		Recommendation	Intraclass Correlation Coefficient
	Scope and Purpose	Stakeholder Involvement	Rigor of Development	Clarity of Presentation	Applicability	Editorial Independence	≥60%	≥30%	Grade (Level)	
Chinese Society of Cardiovascular Anesthesiology Day Surgery Anesthesia Branch (11)	88.89	41.67	27.78	77.78	53.13	50.00	2	6	B	0.817
Bailey CR et al. (7)	87.50	70.83	41.67	94.44	76.04	83.33	5	6	B	
Chinese Society of Anesthesiology (10)	91.67	65.28	32.30	70.83	42.71	54.17	3	3	B	
De Luca et al. (2)	72.22	86.11	80.21	98.61	98.61	70.83	6	6	A	

#### Quality assessment results of the included consensuses

The 3 expert consensuses (15–17) were evaluated for their methodological quality. Notably, the Chinese Society of Anesthesiology (15) was evaluated as “No” for item 6, “Are there any inconsistencies between the proposed viewpoints and previous literature?”, while all other literature items were evaluated as “Yes”. Overall, the quality of expert consensus literature was high, as shown in Table 3.

**Table 3:** Quality assessment results of the included expert consensuses (*n* = 3).

Included Literature	Item 1	Item 2	Item 3	Item 4	Item 5	Item 6
Chinese Society of Anesthesiology (15)	Yes	Yes	Yes	Yes	Yes	No
Chinese Society of Pediatric Surgery Endoscopic Surgery Group (16)	Yes	Yes	Yes	Yes	Yes	Yes
National Clinical Research Center for Geriatric Diseases (17)	Yes	Yes	Yes	Yes	Yes	Yes

Item 1: Is the source of the viewpoints clearly indicated?

Item 2: Do the viewpoints come from experts in the field?

Item 3: Are the proposed viewpoints centered on the interests of the relevant population being studied?

Item 4: Are the conclusions based on thorough analysis, and are they logically and clearly presented?

Item 5: Are the references to existing literature properly and accurately cited?

Item 6: Are there any inconsistencies between the proposed viewpoints and previous literature?

#### Quality assessment results of the included meta-analyses and systematic reviews

The 4 meta-analyses and systematic reviews (18–21) were evaluated using the AMSTAR2 tool, based on the 2017 Joanna Briggs Institute (JBI) guidelines, to assess the methodological quality. The study by Liu SH et al. (18) was rated as moderate quality, while the remaining studies were rated as high quality. The overall quality of the meta-analyses and systematic reviews was high, and all were included in the study.

### Evidence summary

This project summarized 26 best evidence items across three major themes: the construction of day surgery systems, quality and safety, and perioperative management. Full details of these evidence items can be found in Table 4.

**Table 4:** Summary of best evidence for perioperative management of pediatric Day surgery.

Topic		Evidence Content	Level of Evidence	Grade of Recommendation
Constructing a Day Surgery System	Medical Resources	1. To implement day surgery, it is necessary to equip fixed day surgery operating rooms with essential equipment and facilities, post-anesthesia recovery rooms, and medical beds that meet the requirements of day surgery (10, 11, 16, 17)	1b	A
		2. Close collaboration between experienced surgeons and anesthesiologists is required; nurses with strong professional communication skills are needed to provide preoperative care, postoperative care, and follow-up (10, 11, 16).	1b	A
		3. A general dispatcher position should be established to coordinate various departments, such as the day surgery ward and operating room, to comprehensively plan and coordinate the operation of the day surgery room (7, 17).	5b	B
		4. Day surgery rooms should reasonably allocate medical auxiliary personnel, implementing standardized work processes to regulate behavior and improve service quality and operational efficiency (17).	5b	B
Quality and Safety	Management Regulations	5. Ensure the establishment of a 24-hour emergency response system (10, 11, 16, 17)	1b	A
		6. Medical institutions should establish work systems related to day surgery services and develop reasonable clinical pathways that include training systems for medical staff, management systems for qualified anesthesiologists and surgeons, patient selection for day surgery, preoperative assessment, anesthesia methods, surgical methods, postoperative recovery, follow-up procedures, emergency plans for day surgery, and medical record management systems (10, 16, 17).	1b	A
	Monitoring Indicators	7. Utilizing quality and safety monitoring indicators, including no-show rate, same-day surgery cancellation rate, unplanned surgery delay rate, unplanned delayed discharge rate, unplanned readmission rate, unplanned reoperation rate, unplanned referral rate, postoperative complications and adverse reactions, and satisfaction of pediatric patients and their families (7, 16, 19).	1a	B
		8. During the surgical process, monitoring indicators such as on-time start rate, surgical safety checklist, surgical item count, and surgical pathology specimen submission must be used to supervise quality (16).	1b	A
Comprehensive Process Management	Preoperative Assessment	9. Anesthesiologists and surgeons jointly should collaborate to assess and screen patients suitable for day surgery (7, 10, 11, 13, 16).	1a	A
		10. The assessment content includes medical history, physical examination, and auxiliary examinations. The preoperative examinations should be selected based on the child's condition, surgical method, and anesthesia method, and should be consistent with the examination items for routine inpatient children (10, 11, 15, 16).	1b	A
		11. A dedicated preoperative anesthesiology clinic should be established, and pediatric day surgery patients need to visit the anesthesiology clinic before surgery (10, 11, 15)	1b	A
		12. Children suitable for day surgery and anesthesia should typically meet the following criteria:The recommended age range for pediatric day surgery is from 3 months to 18 years, with premature infants requiring a post-conceptual age of over 60 weeks (7, 10, 11, 14); classified as American Society of Anesthesiologists (ASA) class I, II, or certain class III patients without significant cardiopulmonary disease or preoperative contraindications for surgery (2, 7, 10, 14); For children with preoperative acute, simple upper respiratory tract infections such as runny nose, cough, or fever, it is recommended to schedule the surgery 1 week after the symptoms disappear. However, if symptoms affecting the lower respiratory tract occur, surgery should be postponed for at least 1 month after the child's recovery (2, 16); Family members capable of perioperative care should accompany the child, have accessible contact information, and be available for follow-up and emergency handling (2, 7, 11, 16).	5b	A
	Surgical Arrangement	13. Surgical scheduling is one of the key elements for the efficient operation of day surgery wards. The process should involve (7, 16, 17, 19): (1) doctors and children's parents propose surgical appointment dates and requirements; (2) anesthesiologists assess and issue day surgery anesthesia assessment forms; (3) doctors and nurses from the day surgery department review the admission criteria and organize the overall schedule; and (4) appointment information is well communicated among medical staff, nursing staff, and the patient's family.	1a	A
		14. Surgical scheduling needs to prioritize the surgical method and the necessary postoperative recovery time for patients in the ward (17, 18).	1b	B
	Health Education	15. Preoperative education must include oral and written instructions given to children and their guardians before surgery (7, 10, 11, 14).	1a	A
		16. A variety of educational methods should be employed, including oral education, printed materials, videos, pictures, and internet platforms such as WeChat to ensure that both the children and families understand the procedure and can cooperate fully with the treatment plan (7, 14, 16, 18–21).	1a	A
		17. The preoperative education content should include several key elements, including (1) day surgery ward admission process; (2) surgical and anesthesia methods, risks, complications, and contingency plans; and (3) preparation for children before admission, such as precautions before day surgery, key points for cooperation during hospitalization, knowledge related to rapid recovery, surgical psychological stress intervention, infection prevention, medication plan, and the role of family accompaniment (10, 11, 14, 18).	2c	A
	Surgery Day Management	18. Pediatric day surgery patients need to fast from solid food for 6−8 h, formula milk for 6 h, breast milk for 4 h, and clear liquids for 2 h (7, 10, 11, 14–16, 18).	1a	A
		19. Staggered admission can be implemented to enhance operational efficiency, with two admission times, in the morning and afternoon (7).	5b	B
		20. Anesthesia for pediatric day surgery must meet surgical requirements while also facilitating rapid postoperative recovery for the child (7, 11, 14, 15).	1b	A
		21. The selection of anesthetic drugs should be based on criteria such as rapid onset, quick elimination, short duration of action, good analgesic and sedative effects, minimal impact on cardiopulmonary function, and absence of significant adverse reactions or discomfort (7, 11, 14, 15).	1b	A
	Postoperative Care and Education	22. Routine pain assessment should be conducted, and postoperative pain management should adopt early, aggressive, multimodal analgesic therapy, minimizing the use of opioids as much as possible (7, 11–15).	1a	A
		23. Postoperative care should include (1) close observation of the patient's condition and timely treatment to reduce complications; (2) once the child is awake, they should be allowed to adopt a free and comfortable position, with parents holding infants and young children to reduce crying and vomiting to prevent aspiration; (3) after the child is fully awake, they can be given water to drink, with the diet transitioning from liquid to semi-liquid and then to a normal diet; (4) the surgical wound should be carefully monitored for any bleeding after surgery; and (5) family members should be trained and educated to improve their nursing skills and ability to care for the child at home (11, 16).	1a	A
		24. Discharge criteria after day surgery and anesthesia should be strictly followed, with both the anesthesiologist and surgeon jointly evaluating the child's condition before discharge (10, 11, 15, 16).	1a	A
		25. Discharge guidance and education should be provided to the child's parents, informing them of basic postoperative care knowledge and precautions, and providing them with the day surgery center's contact number for emergencies (11, 16).	1a	A
		26. It is recommended to follow up with day surgery patients within 24 h after discharge to promptly identify and manage any anesthesia- or surgery-related complications (10, 11, 15, 16).	1b	A

## Discussion

In this synthesis, 26 best-evidence recommendations were organized into three operational domains—construction of day surgery systems, quality and safety, and comprehensive perioperative management—providing an implementation-facing roadmap for pediatric day-surgery teams (Table 4). Rather than restating each recommendation, this Discussion interprets how the domains address common perioperative risks and operational gaps across different healthcare settings.

### Establishing a pediatric Day surgery system as the foundation for safe medical practices

Evidence items 1–4 indicate that safe pediatric day surgery depends on a team-based model supported by appropriate staffing structures. Close collaboration between experienced surgeons and anesthesiologists (evidence item 2) is central to consistent eligibility decisions, perioperative risk management, and discharge readiness. This collaboration is operationalized through joint assessment at entry and joint evaluation before discharge (evidence items 9 and 24), reducing the risk of single-discipline decision bias.

Beyond core clinicians, evidence items 3–4 highlight the value of defined roles for scheduling/coordination and auxiliary staff to support workflow continuity and efficiency. Although the evidence level is lower for these items, clearly assigning responsibilities and standardizing work processes can reduce avoidable delays and facilitate smoother transitions across perioperative stages in high-throughput day-surgery settings.

Infrastructure and recovery capability remain essential prerequisites. Evidence item 1 supports equipping fixed day-surgery operating rooms with appropriate facilities, PACU capability, and beds meeting day-surgery requirements, aligning system capacity with the rapid turnover and close monitoring needs of pediatric patients. Finally, evidence item 5 underscores that a 24-hour emergency response and escalation system is a safety baseline for pediatric day surgery, spanning both in-hospital emergencies and post-discharge contingencies.

### Improving systems, strengthening supervision, and promoting safe and smooth implementation of pediatric Day surgery

Evidence items 6–8 emphasize that pediatric day surgery benefits from standardized institutional systems and continuous quality supervision. Establishing clinical pathways and staff training/authorization systems (evidence item 6), alongside process and outcome monitoring (evidence items 7–8), supports continuous quality improvement. In practice, information systems can facilitate tracking indicators such as no-show and cancellation rates, unplanned delays, delayed discharge, readmission, complications/adverse reactions, satisfaction, and checklist adherence (evidence item 7), enabling audit-and-feedback cycles for pathway refinement.

In developing settings undergoing rapid scale-up, these monitoring functions are particularly valuable because bottlenecks (e.g., preoperative readiness, scheduling mismatches, and post-discharge uncertainty) may emerge quickly with increasing volume. Digital health education and child-friendly perioperative preparation tools described in the broader literature (22, 23) can complement the evidence-based pathway by improving caregiver understanding, reducing anxiety, and supporting adherence to perioperative instructions. Research has demonstrated that implementing gamified health intervention information systems throughout the entire pediatric perioperative period is effective (22).

#### Comprehensive perioperative management of children undergoing Day surgery to ensure safe and effective surgery and rapid recovery

Evidence items 9–26 provide full-process perioperative management across preoperative assessment, surgical arrangement, day-of-surgery management, postoperative care, and education. A key implication is that front-loading eligibility and readiness assessment can prevent downstream disruptions. Joint surgeon–anesthesiologist screening (evidence item 9), structured assessment content (evidence item 10), and preoperative anesthesiology clinic evaluation (evidence item 11) together form an upstream safety filter. Although most children can safely undergo day surgery, the same-day cancellation rate for pediatric day surgery is significantly higher than that for adults, approaching 10%, and is primarily attributed to pathogenic factors, particularly upper respiratory tract infections (19, 24, 25). This finding is highly relevant to clinical practice in China, where the high incidence and rapid transmission of respiratory infections—due to children's communal living environments and immune system characteristics—have become a major barrier to the efficiency of day surgery implementation. Interestingly, a recent study from the United States reported a pediatric day surgery cancellation rate of only 3.8% (26), significantly lower than clinical observation data in China. More importantly, cancellation rates in the US showed distinct disparities based on social determinants: children from affluent neighborhoods had the lowest cancellation rates, while those from vulnerable groups faced the highest risk; notably, Black children had a 1.48 times higher risk of cancellation than White children. This comparison highlights substantial differences in the core challenges faced by different healthcare systems.

For infection-related cancellations, implementation emphasis should prioritize: clear timing guidance for URTI/LRTI-related postponement within eligibility criteria (evidence item 12), structured pre-admission screening and caregiver preparation (evidence items 15–17), and scheduling/throughput management that reduces crowding and supports readiness (evidence items 13 and 19). For disparity-related barriers reported in some settings (e.g., variation by neighborhood opportunity and race/ethnicity) (2), implementation emphasis may differ: accessible multimodal education and reminders (evidence items 15–17), standardized pathways for scheduling and communication (evidence item 13), and reliable post-discharge follow-up and contact mechanisms (evidence items 25–26) may reduce preventable cancellations by improving readiness and continuity.

Finally, postoperative safety in pediatric day surgery depends on clear discharge criteria (evidence item 24), explicit discharge guidance with emergency contact information (evidence item 25), and early follow-up (evidence item 26). Together, these components help close the “post-discharge safety loop,” supporting timely identification and management of complications in the first 24 h after discharge.

##### Strengths and limitations

Methodological quality was appraised using AGREE II and JBI critical appraisal tools, supported by a comprehensive search across multiple databases and bilingual (Chinese/English) sources. The independent 17-expert panel strengthened clarity and contextual usability through language/terminology validation of translated Chinese wording only, without influencing evidence appraisal or grading. Limitations include language restriction and heterogeneity across healthcare systems, which may affect direct generalizability and necessitate local adaptation, especially under resource constraints.

### Practical implications

This evidence synthesis translates 26 recommendations into a practical roadmap for pediatric day-surgery teams (Table 4). First, institutions should establish a multidisciplinary model in which surgeons, anesthesiologists, and specialized nurses coordinate the full perioperative pathway, with joint eligibility screening and discharge evaluation as key safety checkpoints (evidence items 9 and 24).

Second, we recommend implementing an information-based preoperative evaluation and education system, defined as a structured, data-supported workflow that integrates pre-admission questionnaires, standardized assessment templates, automated risk stratification and reminders, and tailored caregiver education packages (e.g., checklists, short videos, and procedure-specific instructions). Such systems can improve preoperative readiness and communication across teams and families, thereby reducing preventable same-day cancellations and improving operational efficiency (evidence items 7, 13, and 15–17).

Third, continuous staff training should be combined with regular quality and safety monitoring. Training should focus on updated pathways/guidelines, perioperative communication, discharge teaching, and emergency escalation procedures, delivered through simulations/workshops, online modules, and periodic competency reviews (evidence item 6). Quality monitoring should track key indicators such as no-show and cancellation rates, unplanned delays, delayed discharge, readmission, complications/adverse reactions, satisfaction, and checklist adherence, supported by dashboards and audit-feedback processes to drive continuous improvement (evidence items 7–8).

### Limitations

This study has several limitations that should be acknowledged. First, by including only literature in Chinese and English, we may have introduced a language bias, potentially omitting relevant studies from other regions. Second, the reliance on published literature, including guidelines and expert consensuses, may be subject to publication bias, where studies with positive findings are more likely to be reported. Third, only one randomized controlled trial was identified; therefore, several recommendations rely on guidelines, systematic reviews, heterogeneous evidence, and expert consensus, and may require local adaptation. Finally, while this synthesis provides a robust framework, heterogeneity among global healthcare systems may limit direct generalizability of certain recommendations, requiring adaptation to local resources and care pathways.

## Conclusions

This review synthesized 26 best-evidence recommendations and organized them into three operational domains (Table 4), providing an implementation-facing roadmap for pediatric day-surgery teams. Because implementation capacity varies across settings, we highlight three high-impact priorities for rapid adoption: (1) front-load a joint surgeon–anesthesiologist eligibility assessment; (2) standardize caregiver preparation using checklists and multimodal education; and (3) close the post-discharge safety loop within 24 h through nurse-led follow-up (Table 4). Collectively, these priorities offer a feasible staged pathway to strengthen safety, efficiency, and family experience while allowing local adaptation.
