# Supplementary material for: Perioperative management in pediatric day surgery: a synthesis of best evidence
Source: Front Pediatr. 2026 Mar 27;14:1741673. doi: 10.3389/fped.2026.1741673 (PMC13066185; doi:10.3389/fped.2026.1741673)
Supplement: Supplementary file 1 [file Table1.docx]

Supplementary Table 1: General Characteristics of Included Literature (n=15)

| Included Literature | Literature Source | Literature Type | Literature Topic | Publication/ Update Year |
| --- | --- | --- | --- | --- |
| Schechter W. [21] | UpToDate | Clinical Decision | Management of acute perioperative pain in infants and children | 2024 |
| Shapiro FE et al.[22] | UpToDate | Clinical Decision | Anesthesia outpatient clinic | 2024 |
| SA Black et al.[23] | UpToDate | Clinical Decision | Drugs and techniques for general anesthesia in neonates and children | 2024 |
| Chinese Society of Cardiovascular Anesthesiology Day Surgery Anesthesia Branch[19] | MedSci | Guideline | Pediatric day surgery anesthesia guideline | 2019 |
| CR Bailey[20] | American Academy of Pediatrics | Guideline | 2019 Day Surgery Guideline | 2019 |
| Chinese Society of Anesthesiology[18] | VIP Database | Guideline | Day surgery anesthesia guideline | 2023 |
| de Luca et al[2] | Italian Society of Pediatric Surgery | Guideline | Italian Society of Pediatric Surgery and Pediatric Anesthesiology Society pediatric day surgery guideline | 2018 |
| Chinese Society of Anesthesiology[24] | CNKI | Expert Consensus | Day surgery anesthesia expert consensus | 2016 |
| Chinese Society of Pediatric Surgery Endoscopic Surgery Group et al[25] | VIP Database | Expert Consensus | Pediatric surgery day surgery expert consensus | 2020 |
| National Clinical Research Center for Geriatric Diseases et al[26] | VIP Database | Expert Consensus | Chinese expert consensus on operation and management of day surgery units in general hospitals | 2022 |
| Liu Shihui et al.[27] | Wanfang Database | Systematic Review | Meta-analysis of the effectiveness of enhanced recovery nursing in reducing postoperative complications in pediatric day surgery in China | 2023 |
| Liu Qian et al.[28] | CNKI | Systematic Review | Meta-analysis of temporary cancellation rates in day surgery | 2024 |
| Rantala A et al.[29] | PubMed | Systematic Review | Effectiveness of web-based mHealth interventions in pediatric outpatient surgery: A systematic review and meta-analysis of randomized controlled trials | 2020 |
| Kerimaa H et al.[30] | PubMed | Systematic Review | Effectiveness of preoperative interventions for day surgery in preschool children and their parents: A systematic review and meta-analysis of randomized controlled trials | 2023 |
| Akkoyun S et al.[17] | PubMed | Randomized Controlled Trial | Effect of perioperative written materials on anxiety levels of parents and home care of pediatric outpatient surgery patients: A randomized controlled trial | 2023 |

Supplementary Table 2: Quality Evaluation of the Included Guidelines using AGREE II (n=4)

| Included Literature | Standardized Percentage for Each Domain (%) | | | | | | Overall Assessment (n) | | Recommendation | Intraclass Correlation Coefficient |
| --- | --- | --- | --- | --- | --- | --- | --- | --- | --- | --- |
|  | Scope and Purpose | Stakeholder Involvement | Rigor of Development | Clarity of Presentation | Applicability | Editorial Independence | ≥60% | ≥30% | Grade (Level) |  |
| Chinese Society of Cardiovascular Anesthesiology Day Surgery Anesthesia Branch [19] | 88.89 | 41.67 | 27.78 | 77.78 | 53.13 | 50.00 | 2 | 6 | B | 0.817 |
| Bailey CR et al. [20] | 87.50 | 70.83 | 41.67 | 94.44 | 76.04 | 83.33 | 5 | 6 | B |  |
| Chinese Society of Anesthesiology[18] | 91.67 | 65.28 | 32.30 | 70.83 | 42.71 | 54.17 | 3 | 3 | B |  |
| De Luca et al.[2] | 72.22 | 86.11 | 80.21 | 98.61 | 98.61 | 70.83 | 6 | 6 | A |  |

Note: AGREE II, Appraisal of Guidelines for Research & Evaluation II. The recommendation grade is based on the AGREE II evaluation. Grade A: Strongly recommended; Grade B: Recommended, but with modifications or considerations.

Supplementary Table 3: Quality Assessment of the Included Expert Consensuses (n=3)

| Included Literature | Item 1 | Item 2 | Item 3 | Item 4 | Item 5 | Item 6 |
| --- | --- | --- | --- | --- | --- | --- |
| Chinese Society of Anesthesiology[24] | Yes | Yes | Yes | Yes | Yes | No |
| Chinese Society of Pediatric Surgery Endoscopic Surgery Group[25] | Yes | Yes | Yes | Yes | Yes | Yes |
| National Clinical Research Center for Geriatric Diseases[26] | Yes | Yes | Yes | Yes | Yes | Yes |

Note:

Item 1: Is the source of the viewpoints clearly indicated?

Item 2: Do the viewpoints come from experts in the field?

Item 3: Are the proposed viewpoints centered on the interests of the relevant population being studied?

Item 4: Are the conclusions based on thorough analysis, and are they logically and clearly presented?

Item 5: Are the references to existing literature properly and accurately cited?

Item 6: Are there any inconsistencies between the proposed viewpoints and previous literature?

Supplementary Table 4: Summary of Best Evidence for Perioperative Management of Pediatric Day Surgery

| Topic | | Evidence Content | Level of Evidence | Grade of Recommendation |
| --- | --- | --- | --- | --- |
| Constructing a Day  Surgery  System | Medical Resources | 1. To implement day surgery, it is necessary to equip fixed day surgery operating rooms with essential equipment and facilities, post-anesthesia recovery rooms, and medical beds that meet the requirements of day surgery[18, 19, 25, 26]. | 1b | A |
|  |  | 2. Close collaboration between experienced surgeons and anesthesiologists is required; nurses with strong professional communication skills are needed to provide preoperative care, postoperative care, and follow-up[18, 19, 25]. | 1b | A |
|  |  | 3. A general dispatcher position should be established to coordinate various departments, such as the day surgery ward and operating room, to comprehensively plan and coordinate the operation of the day surgery room[20, 26]. | 5b | B |
|  |  | 4. Day surgery rooms should reasonably allocate medical auxiliary personnel, implementing standardized work processes to regulate behavior and improve service quality and operational efficiency[26]. | 5b | B |
|  |  | 5. Ensure the establishment of a 24-hour emergency response system[18, 19, 25, 26]. | 1b | A |
| Quality and Safety | Management Regulations | 6. Medical institutions should establish work systems related to day surgery services and develop reasonable clinical pathways that include training systems for medical staff, management systems for qualified anesthesiologists and surgeons, patient selection for day surgery, preoperative assessment, anesthesia methods, surgical methods, postoperative recovery, follow-up procedures, emergency plans for day surgery, and medical record management systems[18, 25, 26]. | 1b | A |
|  | Monitoring Indicators | 7. Utilizing quality and safety monitoring indicators, including no-show rate, same-day surgery cancellation rate, unplanned surgery delay rate, unplanned delayed discharge rate, unplanned readmission rate, unplanned reoperation rate, unplanned referral rate, postoperative complications and adverse reactions, and satisfaction of pediatric patients and their families[20, 25, 28]. | 1a | B |
|  |  | 8. During the surgical process, monitoring indicators such as on-time start rate, surgical safety checklist, surgical item count, and surgical pathology specimen submission must be used to supervise quality[25]. | 1b | A |
| Comprehensive Process Management | Preoperative  Assessment | 9. Anesthesiologists and surgeons jointly should collaborate to assess and screen patients suitable for day surgery[18-20, 22, 25]. | 1a | A |
|  |  | 10. The assessment content includes medical history, physical examination, and auxiliary examinations. The preoperative examinations should be selected based on the child's condition, surgical method, and anesthesia method, and should be consistent with the examination items for routine inpatient children[18, 19, 23-25]. | 1b | A |
|  |  | 11. A dedicated preoperative anesthesiology clinic should be established, and pediatric day surgery patients need to visit the anesthesiology clinic before surgery[18, 19, 24] | 1b | A |
|  |  | 12. Children suitable for day surgery and anesthesia should typically meet the following criteria:  The recommended age range for pediatric day surgery is from 3 months to 18 years, with premature infants requiring a post-conceptual age of over 60 weeks[18-20, 23]; classified as American Society of Anesthesiologists (ASA) class I, II, or certain class III patients without significant cardiopulmonary disease or preoperative contraindications for surgery[2, 18, 20, 23]; For children with preoperative acute, simple upper respiratory tract infections such as runny nose, cough, or fever, it is recommended to schedule the surgery 1 week after the symptoms disappear. However, if symptoms affecting the lower respiratory tract occur, surgery should be postponed for at least 1 month after the child's recovery[2, 25]; Family members capable of perioperative care should accompany the child, have accessible contact information, and be available for follow-up and emergency handling[2, 19, 20, 25]. | 5b | A |
|  | Surgical Arrangement | 13. Surgical scheduling is one of the key elements for the efficient operation of day surgery wards. The process should involve[20, 25, 26, 28]: (1) doctors and children's parents propose surgical appointment dates and requirements; (2) anesthesiologists assess and issue day surgery anesthesia assessment forms; (3) doctors and nurses from the day surgery department review the admission criteria and organize the overall schedule; and (4) appointment information is well communicated among medical staff, nursing staff, and the patient’s family. | 1a | A |
|  |  | 14. Surgical scheduling needs to prioritize the surgical method and the necessary postoperative recovery time for patients in the ward[26, 27]. | 1b | B |
|  | Health Education | 15. Preoperative education must include oral and written instructions given to children and their guardians before surgery[18-20, 23, 31]. | 1a | A |
|  |  | 16. A variety of educational methods should be employed, including oral education, printed materials, videos, pictures, and internet platforms such as WeChat to ensure that both the children and families understand the procedure and can cooperate fully with the treatment plan[20, 23, 25, 27, 29, 30]. | 1a | A |
|  |  | 17. The preoperative education content should include several key elements, including (1) day surgery ward admission process; (2) surgical and anesthesia methods, risks, complications, and contingency plans; and (3) preparation for children before admission, such as precautions before day surgery, key points for cooperation during hospitalization, knowledge related to rapid recovery, surgical psychological stress intervention, infection prevention, medication plan, and the role of family accompaniment[18, 19, 23, 27]. | 2c | A |
|  | Surgery Day Management | 18. Pediatric day surgery patients need to fast from solid food for 6-8 hours, formula milk for 6 hours, breast milk for 4 hours, and clear liquids for 2 hours[18-20, 23-25, 27]. | 1a | A |
|  |  | 19. Staggered admission can be implemented to enhance operational efficiency, with two admission times, in the morning and afternoon[20]. | 5b | B |
|  |  | 20. Anesthesia for pediatric day surgery must meet surgical requirements while also facilitating rapid postoperative recovery for the child[19, 20, 23, 24]. | 1b | A |
|  |  | 21. The selection of anesthetic drugs should be based on criteria such as rapid onset, quick elimination, short duration of action, good analgesic and sedative effects, minimal impact on cardiopulmonary function, and absence of significant adverse reactions or discomfort[19, 20, 23, 24]. | 1b | A |
|  | Postoperative Care and Education | 22. Routine pain assessment should be conducted, and postoperative pain management should adopt early, aggressive, multimodal analgesic therapy, minimizing the use of opioids as much as possible[19-24]. | 1a | A |
|  |  | 23. Postoperative care should include (1) close observation of the patient's condition and timely treatment to reduce complications; (2) once the child is awake, they should be allowed to adopt a free and comfortable position, with parents holding infants and young children to reduce crying and vomiting to prevent aspiration; (3) after the child is fully awake, they can be given water to drink, with the diet transitioning from liquid to semi-liquid and then to a normal diet; (4) the surgical wound should be carefully monitored for any bleeding after surgery; and (5) family members should be trained and educated to improve their nursing skills and ability to care for the child at home[19, 25]. | 1a | A |
|  |  | 24. Discharge criteria after day surgery and anesthesia should be strictly followed, with both the anesthesiologist and surgeon jointly evaluating the child’s condition before discharge[18, 19, 24, 25]. | 1a | A |
|  |  | 25. Discharge guidance and education should be provided to the child's parents, informing them of basic postoperative care knowledge and precautions, and providing them with the day surgery center's contact number for emergencies[19, 25]. | 1a | A |
|  |  | 26. It is recommended to follow up with day surgery patients within 24 hours after discharge to promptly identify and manage any anesthesia- or surgery-related complications[18, 19, 24, 25]. | 1b | A |

Note: Level of Evidence (LoE) is based on the Joanna Briggs Institute (JBI) Evidence-Based Healthcare Model (2014): 1a-Systematic review of RCTs; 1b-Systematic review of RCTs and other study designs; 2c-Systematic review of qualitative studies; 5b-Expert consensus. Grade of Recommendation (GoR) is based on the JBI grading system: Grade A-Strong recommendation; Grade B-Weak recommendation. Abbreviation: ASA, American Society of Anesthesiologists.
